# Case report: Complete response after transcatheter arterial chemoembolization combined with donafenib plus tislelizumab therapy for hepatocellular carcinoma with main trunk portal vein tumor thrombus in a patient coinfected with HIV and HBV

**DOI:** 10.3389/fimmu.2024.1422801

**Published:** 2024-07-15

**Authors:** Xuhua Xiao, Haixiao Fu, Huixia Qin, Longkuan Xu, Jing Gu, Zhan Zhang, Houxiang Ya, Kaiwen Jiang, Zhiyuan Jian, Shuqun Li

**Affiliations:** ^1^ Department of Gastroenterology, Affiliated Hospital of Guilin Medical University, Guilin, Guangxi, China; ^2^ Department of pathology, Affiliated Hospital of Guilin Medical University, Guilin, Guangxi, China; ^3^ Interventional Center, Affiliated Hospital of Guilin Medical University, Guilin, Guangxi, China; ^4^ Department of Hepatobiliary Pancreatic Surgery, Affiliated Hospital of Guilin Medical University, Guilin, Guangxi, China; ^5^ Department of Gastrointestinal Surgery, Affiliated Hospital of Guilin Medical University, Guilin, Guangxi, China

**Keywords:** hepatocellular carcinoma, main trunk portal vein thrombus, donafenib, tislelizumab, HIV, complete response

## Abstract

**Background:**

Coinfection with the human immunodeficiency virus (HIV) and the hepatitis B virus (HBV) occurs in 5–67% of patients with HIV. HIV weakens the human immune system and leads to various tumors. Patients with unresectable hepatocellular carcinoma (HCC) and HIV experience poor treatment efficacy and have a short survival period. Approximately 70% of cases of HCC are diagnosed at advanced stages due to the subtle onset of the disease. As a result, most cases are not suits for curative therapy. Transcatheter arterial chemoembolization (TACE) is the first-line treatment for intermediate-stage HCC and is commonly used to treat unresectable HCC in China. Recent advancements in systemic treatments have significantly enhanced the effectiveness of unresectable HCC treatment. Several previous study showed that combination treatment combination therapy can enhance the efficacy. Notably, studies proposed that TACE combined targeted drugs with immune checkpoint inhibitors results in a high objective response rate and overall survival. However, the novelty of this study lies in its report of a complete response using a triple combination in patients with HIV and HCC with main trunk portal vein tumor thrombus.

**Case presentation:**

A 57-year-old woman was diagnosed with HCC with a main trunk portal vein tumor thrombus combined with HIV infection, cirrhosis, and chronic viral hepatitis. She underwent TACE and was administered donafenib and tislelizumab. This triple therapy treatment regimen resulted in a clinical complete response according to the modified Response Evaluation Criteria in Solid Tumors (mRECIST) based on contrast-enhanced computed tomography.

**Conclusion:**

We first used TACE combined with donafenib and tislelizumab for HCC patients with main trunk portal vein tumor thrombus and HIV-HBV coinfection and achieved complete response.

## 1 Introduction

A significant proportion of patients with human immunodeficiency virus (HIV) infection are coinfected with hepatitis B virus (HBV) due to common routes of transmission (1, 2). Acquired human immunodeficiency disease (AIDS), caused by HIV infection, results in partial or complete loss of immune function, rendering individuals susceptible to various opportunistic infections or tumors (3, 4). Though the number of novel infections per year has decreased recently in China, the annual number of new infections has increased in other regions including Central Asia, Eastern Europe, and North Africa (5). Advances in medical technology have significantly prolonged the survival of patients with HIV, increasing the risk of developing malignant tumors, and an increasing proportion of patients with HIV/AIDS have been diagnosed with hepatocellular carcinoma (HCC). The incidence of HCC in patients with AIDS is 7.7 times higher than that in the general population. Patients with HIV and HCC have a poorer survival prognosis than HIV-negative patients with HCC (6).

HCC poses a significant challenge on a global scale, ranking sixth and third in terms incidence and mortality, respectively, among malignant tumors (7). Approximately 70% of cases of HCC are diagnosed at advanced stages due to the subtle onset of the disease; therefore, most patients are not candidates for curative interventions, such as surgery, ablation, or liver transplantation. Portal vein tumor thrombus (PVTT) is detected in 10–60% of patients with HCC and is associated with a median survival of 2.7–4.0 months (8). HCC patients who are affected by PVTT, especially main trunk portal vein thrombus (Vp4) have extremely advanced disease that is considered unresectable.

HCC combined with PVTT often requires comprehensive therapy because of its low surgical resection rate and high recurrence rate. Transcatheter arterial chemoembolization (TACE) is the first-line therapy for intermediate-stage HCC and has been extensively used to treat unresectable HCC (uHCC) in China (9), though HCC with PVTT is considered a contraindication for TACE (10). However, several recent studies reported that patients with good hepatic function and sufficient collateral circulation around the thrombus may tolerate and benefit from TACE (11–13).

Recent progress in systematic therapy, especially the progress on targeted agencies and immunotherapy, the primary treatment for uHCC has improved the treatment of uHCC significantly. Donafenib, a novel inhibitor that targets multiple kinases, is superior to sorafenib in terms of improving overall survival (OS), tolerability, and safety in patients with uHCC, leading to its approval as a first-line therapy (14). Immune checkpoint inhibitors, such as monoclonal antibodies (mAbs) against programmed death receptor-1 (PD-1), programmed death ligand 1 (PD-L1), and cytotoxic T-lymphocyte antigen-4, enhance immunity by targeting inhibitory pathways and facilitating the treatment of uHCC. In a phase III RATIONALE-301 randomized clinical study, tislelizumab, an anti-human PD-1 immunoglobulin G4 mAb, demonstrated comparable efficacy to monotherapy with tislelizumab and sorafenib as a first-line therapy for uHCC based on patient OS. Furthermore, tislelizumab showed an increased objective response rate (ORR), a sustained response, and a favorable safety profile (15). However, the efficacies of these monotherapies are limited.

Various combination strategies have been explored to enhance prognostic outcomes in patients with uHCC. The IMbrave150 trial revealed that the combination of atezolizumab and bevacizumab achieved an ORR of 36% and met the primary endpoints of extended OS and progression-free survival (PFS) with an acceptable safety profile (16). The efficacy of pembrolizumab plus lenvatinib for the treatment of uHCC did not meet the preset endpoint but prolonged the median OS and PFS to 21.2 and 8.2 months, respectively, when compared to treatment with lenvatinib plus placebo in the global LEAP-002 study (17). Moreover, several real-world studies showed that combination therapy could enhance the efficacy, in which the combination of TACE and systemic therapy has shown promising results, especially TACE combined with tyrosine kinase inhibitors (TKIs) and PD-1 inhibitors can significantly increase ORR, OS, and PFS (18–20). Moreover, Zou et al. illustrated that TACE combined lenvatinib and PD-1 inhibitors has more promising clinical outcomes and acceptable safety when compared with TACE plus lenvatinib in patients with HCC and PVTT (21).

HCC is commonly diagnosed in patients with HIV infection; however, there are no reported cases of triple therapy (combining TACE with donafenib and tislelizumab) for the treatment of uHCC with HIV and HBV coinfection. Therefore, this report presents the first case of a patient with HCC with Vp4, HIV infection, HBV infection, cirrhosis who underwent triple therapy. The patient exhibited a significant reduction in tumor size, regression of tumor thrombus, a notable reduction in tumor markers, and prolonged PFS and OS.

## 2 Case report

**Figure 1:**
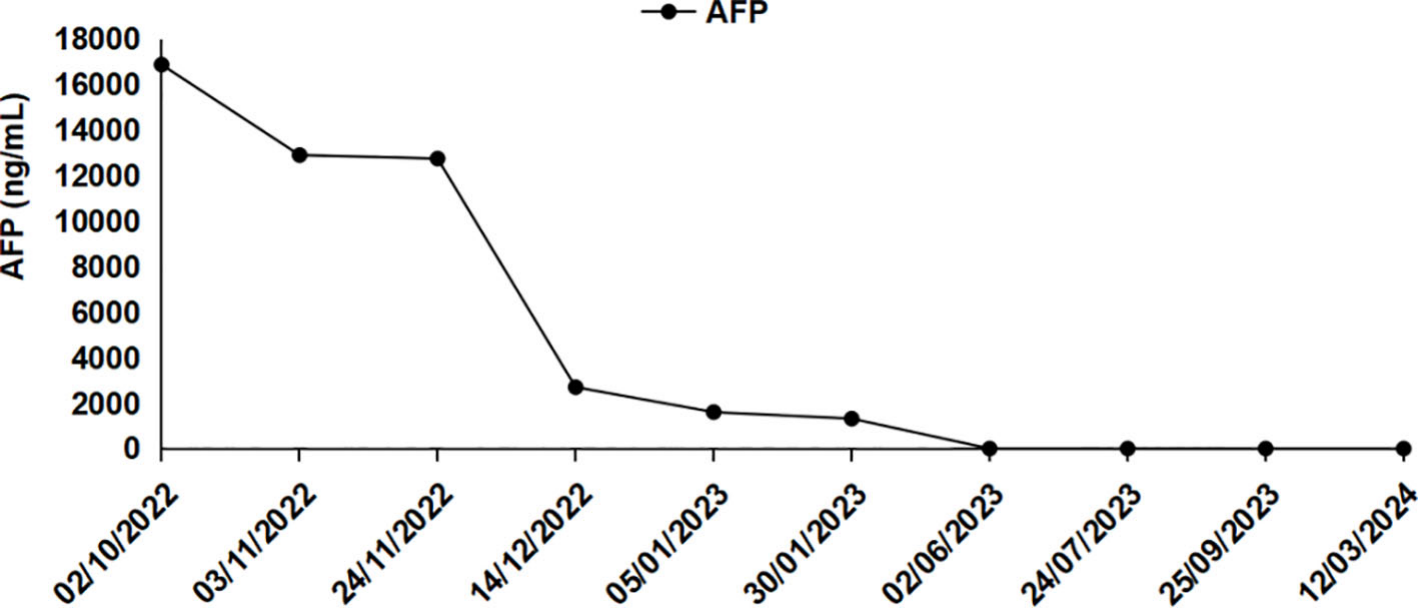
Changes in serum AFP level during clinical treatment (October 2, 2022 for pre-treatment and November 3, 2022 and beyond for post-treatment).

**Figure 2:**
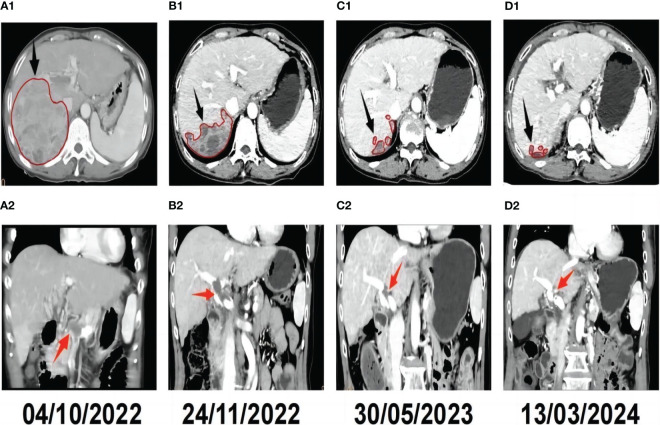
Radiological response evaluation of the liver during the clinical course. Tumor sizes are circled in red.The red arrowhead shows the portal vein tumor thrombus(PVTT), and the black arrowhead shows the hepatic tumor. (**A1)** Pre-treatment contrast-enhanced CT revealed a mass in the right lobe of the liver. **(A2)** Pre-treatment CT showed PVTT. **(B1)** Contrast-enhanced CT revealed that the tumor was necrosis and smaller than pre-treatment 1 month after triple therapy. **(B2)** CT revealed that notable remission of the PVTT 1 month after triple therapy. **(C1)** Complete response (CR) was confirmed by contrast-enhanced CT, which showed no active tumor in the liver seven months after triple therapy. **(C2)** Contrast-enhanced CT revealed that the PVTT was disappear completely seven months after triple therapy. **(D1)** CT obtained 17 months after triple therapy showed that there were still no active or new lesions in the liver. **(D2)** CT obtained 17 months after triple therapy showed that there was no PVTT.

A 57-year-old female farmer was admitted to our hospital on October 1, 2022 due to founding right hepatic occupying lesions for one week. She was 156 cm tall and weighed 53 kg. The patient had a history of HIV at stage A2, with CD4^+^ lymphocytes constituting 23.96% of the T-cell subset and a viral load of 8,000 copies/mL. She also had a history of chronic viral hepatitis B with no prior antiviral therapy. The patient’s HBsAg, anti-HBc, and anti-HBe antibody tests were positive, and the HBV DNA level was significantly high (1190 IU/mL). She had an elevated alpha-fetoprotein (AFP) level of 16,881 ng/mL, the normal value: 0-20 ng/mL (**Figure 1**). Contrast-enhanced computed tomography (CT) revealed multiple hepatic masses within the right hepatic lobe. The largest mass was approximately 100 mm x 95 mm. Tumor thrombus was evident within the right branch and main trunk of the portal vein and hepatic cirrhosis was also observed on CT (**Figures 2, A1-A2**).

The patient was diagnosed with HCC with portal vein tumor thrombus (PVTT), categorized as Vp4 (22). According to the China Liver Cancer Staging guidelines, the tumor was staged as CNCL IIIa, and as Barcelona Clinic Liver Cancer stage C (23, 24). The patient’s liver function was graded as Child-Pugh class B, and she had an Eastern Cooperative Oncology Group Performance Status (ECOG PS) score of 2.

In order to control the replication of the HIV as quickly as possible, on hospital day 3, highly active antiviral therapy (HAART) consisting of indinavir, lamivudine, and stavudine, was initiated based on the patient’s low CD4^+^ lymphocyte ratio after consultation with an infectious disease physician. After multi-disciplinary treatment discussion, the patient was considered to be able to tolerate TACE. On hospital day 4, the patient underwent conventional TACE treatment to reduce tumor burden. Specifically, a 2.7 F microcatheter was inserted through the segmental feeding arteries, and the chemoembolization process was initiated with intra-arterial pirarubicin (20 mg), oxaliplatin (200 mg), and lipiodol (5 mL). Gelatin sponge particles were subsequently injected until a significant reduction in the arterial flow was achieved. Supportive care was administered after TACE. On hospital day 6, the patient began oral donafenib (200 mg twice daily) as a targeted therapy. Tislelizumab (200 mg every three weeks, intravenous) was initiated on hospital day 13.

The patient underwent routine laboratory examinations, including a blood count, coagulation function tests, liver function tests, and a tumor marker analysis. Routine physical examinations were also conducted. Abdominal contrast-enhanced CT was performed every three months to monitor the patient’s condition. Safety assessments were conducted according to the Common Terminology Criteria for Adverse Events Version 5.0, with a focus on treatment-related adverse events.

Approximately one month after treatment, the patient’s AFP level was 12,751 ng/mL (**Figure 1**). The tumor located in the right hepatic lobe was significantly smaller than that observed on pre-treatment CT, with notable remission of the PVTT (**Figures 2, B1-B2**). Seven months post-treatment, during the patient’s third follow-up visit, her AFP level had decreased to 2.71 ng/mL(**Figure 1**). On CT, complete response (CR) was confirmed by contrast-enhanced CT, which showed no active lesions in the liver and the PVTT was disappear completely (**Figures 2, C1-C2**). The patient was considered to have had a clinical complete response based on the mRECIST criteria (25, 26). Seventeen months post-treatment, the patient’s AFP level remained normalized (**Figure 1**), and her ECOG PS score was 0. No new tumor lesions were observed on CT, and there was still no PVTT (**Figures 2, D1-D2**).

After triple therapy with TACE, donafenib, and tislelizumab, the patient experienced diarrhea (grade 2). The patient was treated with loperamide, 8 mg once daily, after colonoscopy ruled out a diagnosis of colitis, and was successfully managed. No notable changes were observed in the patient’s cardiac enzyme levels or thyroid, hepatic, or renal function tests. Throughout the treatment process, excellent tolerability and effectiveness of HAART were observed with undetectable HBV DNA levels.

## 3 Discussion

Combination therapy has significantly improved the outcomes and natural course of patients with uHCC. Locoregional treatments, including TACE, combined with systemic therapy for the treatment of uHCC lead to favorable outcomes. The CHANCE 001 study reported that combining TACE with PD-1/PD-L1 inhibitors and molecular targeted therapy led to increased ORR and significantly prolonged OS and PFS compared to TACE monotherapy for patients with uHCC. According to a retrospective study comparing the therapeutic effect of triple therapy versus dual therapy (TACE plus donafenib) for the treatment of uHCC, a higher disease control rate (96.2% vs. 73.1%, P = 0.021), prolonged median OS (23.1 vs. 14.7 months, P = 0.021), and prolonged median PFS (13.1 vs. 7.2 months, P = 0.017) were achieved in patients who underwent triple therapy compared to those who underwent dual therapy (27). Another multicenter retrospective study reported similar findings (28). A previous study reported that TACE plus lenvatinib and a PD-1 inhibitor were effective and well-tolerated for the treatment of HCC with Vp4, with a median OS of 21.7 months and median PFS of 14.5 months (29). Based on previous studies, we believe that triple therapy may also be effective for HCC with Vp4 in patients coinfected with HIV and HBV. As expected, the current study underscores the remarkable response of triple therapy. However, the patient experienced diarrhea that was controlled under symptomatic treatment. A systematic review showed that the incidence of diarrhea up to 56% when combine immunotherapy with TKIs. Fortunately, the incidence of grade 3–4 events was limited (around 2%) and the risk of severe colitis was low (approximately 0.5%) (30).

HCC treatment in patient with HIV/AIDS is challenging due to the absence of clinical recommendations and lack of clinical experience (31). Moreover, patients with HIV and HBV coinfection have been consistently excluded from clinical studies regarding PD-1 and PD-L1 inhibitors, resulting in limited research regarding the treatment of uHCC combined with HIV infection. However, several studies that evaluate the safety and efficacy of such treatments with the aim of providing more evidence to support their application in patients with both cancer and HIV are ongoing or have recently been completed. This case represents the first report of a patient with HCC with Vp4 and HIV-HBV coinfection who was successfully treated with triple therapy. To the best of our knowledge, this is only the second reported case of complete clinical response of uHCC in a patient with HIV infection. A previous study reported a patient with uHCC and HIV-HCV coinfection who achieved partial response to sorafenib (32). Leonidas et al. reported a patient with HCC with Vp4 who had a complete response after sorafenib (200 mg twice per day) with manageable adverse effects (33).

The exact mechanism for the use of triple therapy for uHCC is unclear, many researchers have sought to explain it. PD-1 serves as the primary inhibitory checkpoint for T-cells, regulating their activation, balancing immunostimulation, and preventing autoimmunity. PD-1 inhibitors block inhibitory signal transmission by activating the PD-1 receptor on T-cells, enhancing cytotoxicity against tumor cells and immune surveillance (34–36). PD-1 inhibitors are also a promising treatment for patients with HIV as they do not induce additional immunosuppression. In a phase I multicenter study of 30 patients with controlled HIV infection and a CD4^+^ T cell count > 100 cells/µL, anti-PD-1 agents were found to be safe (37). Several studies have also indicated no adverse effects on HIV viral load or CD4^+^ T cell count after the use of anti-PD-1 agents (38–42). Tislelizumab, an anti-PD-1 mAb, has been engineered to minimize binding to FcγR on macrophages, avoiding antibody-mediated phagocytosis (43). This mAb stimulates an antitumor immune response and achieves an anticancer effect by restoring T cell activity. Donafenib inhibits various receptor tyrosine kinases and Raf kinases, including those in the RAF/MEK/ERK pathway to impede cancer cell growth and angiogenesis. A potential synergistic mechanism of triple combination therapy for HCC involves TACE inducing tumor tissue necrosis and releasing tumor-specific antigens, which enhances the anti-tumor efficacy of tislelizumab. However, TACE can induce a hypoxic microenvironment by elevating the levels of hypoxia-inducible factor-1, platelet-derived growth factor receptor, and vascular endothelial growth factor, fostering an immunosuppressive tumor microenvironment marked by tumor angiogenesis and progression. Donafenib counteracts tumor angiogenesis by inhibiting angiogenesis and disrupting the nutrient supply to tumor cells. Furthermore, it interferes with crucial signaling pathways within tumor cells, diminishing their proliferation and survival.

Historically, the treatment of HIV patients has evolved since the first case was reported. The first antiretroviral drug, zidovudine (AZT), was approved in 1987, marking a milestone in treatment of HIV infection. After that, a range of antiretroviral drugs in order to target different stages of the virus’s lifecycle, including nucleoside reverse transcriptase inhibitors(NRTIs), non-nucleoside reverse transcriptase inhibitors(NNRTIs), protease inhibitors (PIs), integrase strand transfer inhibitors (INSTIs), fusion inhibitors, and entry inhibitors. The drugs work by targeting key enzymes and processes in the virus replication cycle to prevent or reduce its ability to infect and replicate within host cells. NRTIs mimic natural nucleosides and disrupt the RNA-to-DNA conversion process, while NNRTIs bind to reverse transcriptase to prevent its function. PIs block the protease enzymes that cleave viral proteins, resulting in the production of non-functional virus particles. These drugs, when used in combination, transformed HIV infection into a chronic but manageable disease (44). CD4^+^ helper T lymphocyte count serves as a crucial indicator for assessing the immune status of HIV-infected patients. A decline in CD4^+^ lymphocyte count to 100 cells per liter (normal range: 500-1,600 cells per liter) suggests significant immune exhaustion. According to the World Health Organization on HIV/AIDS (45), a CD4^+^ lymphocyte count below 200 cells per liter is generally considered a marker of AIDS stage (clinical stage III). At this stage, patients experience severe impairment of immune function, predisposing them to opportunistic infections and malignancies. In this patient, the administration of HAART, especially PIs, also demonstrated anticancer effects. These inhibitors can inhibit tumor angiogenesis, invasion, and cell growth and promote apoptosis (33). PIs action is associated with inhibition of phosphatidylinositol 3-kinase (PI3K)/Akt pathway. The possible mechanism is binding to Hsp90 and inhibiting its chaperone function followed by decreased PI3K/Akt signaling (46). PI3K and its downstream kinase Akt regulate various cell processes such as growth, proliferation, survival, migration, apoptosis and hyperactivation (47). Moreover,PI3K/Akt signaling in cancer inhibits promotes activation of mTOR and NF-κB axes that regulate transcription, increase cell growth, survival, proliferation, increase matrix metalloproteinases (MMPs) and vascular endothelial growth factor expression, associated with migration and angiogenesis (48, 49).

## 4 Conclusion

We first used TACE combined with donafenib and tislelizumab for HCC patients with main trunk portal vein tumor thrombus and HIV-HBV coinfection and achieved complete response, highlighting the efficacy of this triple therapy. Further clinical researches are required to validate this treatment strategy and explore the efficacy of triple therapy in different disease conditions and patient populations.

## Data availability statement

The original contributions presented in the study are included in the article/supplementary material. Further inquiries can be directed to the corresponding author/s.

## Ethics statement

The studies involving humans were approved by The Ethics Committee of Affiliated Hospital of Guilin Medical University. The studies were conducted in accordance with the local legislation and institutional requirements. The participants provided their written informed consent to participate in this study. Written informed consent was obtained from the individual(s) for the publication of any potentially identifiable images or data included in this article.

## Author contributions

XX: Conceptualization, Investigation, Writing – original draft, Writing – review & editing. HF: Writing – original draft, Writing – review & editing. HQ: Writing – original draft, Writing – review & editing, Data curation, Visualization. LX: Writing – original draft, Writing – review & editing, Investigation, Software. JG: Writing – review & editing, Data curation, Formal analysis, Writing – original draft. ZZ: Methodology, Project administration, Validation, Writing – review & editing, Writing – original draft. HY: Data curation, Funding acquisition, Validation, Writing – review & editing, Writing – original draft. KJ: Investigation, Methodology, Resources, Writing – review & editing, Writing – original draft. ZJ: Supervision, Validation, Writing – original draft, Writing – review & editing. SL: Formal analysis, Validation, Visualization, Writing – original draft, Writing – review & editing.
